# In vitro anti-yeast activity, kinetics and mechanism of action of essential oils from two cameroonian medicinal plants

**DOI:** 10.1186/s12906-022-03827-3

**Published:** 2023-04-12

**Authors:** Flore Tatiana Kemegni Tchinang, Florentine Marie-Chantal Ndoyé Foé, Rodrigue Keumoe, Elisabeth Menkem Zeuko’o, Fabrice Boyom Fekam, François-Xavier Etoa

**Affiliations:** 1 Department of Biochemistry, Laboratory of Phyto-Biochemistry and Medicinal Plant Studies, Faculty of Science, University of Yaoundé I, P.O. Box 812, Yaoundé, Cameroon; 2 Department of Microbiology, Laboratory of Microbiology, Faculty of Science, University of Yaoundé I, P.O. Box 812, Yaoundé, Cameroon; 3 Laboratory of Phyto-Biochemistry and Medicinal Plant Studies, Antimicrobial and Biocontrol Agents Unit, Faculty of Science, University of Yaoundé I, P.O. Box 812, Yaoundé, Cameroon; 4 Department of Biomedical Sciences, Faculty of Health Sciences, University of Buea, P.O. Box 63, Buea, Cameroon

**Keywords:** *Drypetes gossweileri*, *Pentadiplandra brazzeana*, Essential oils, Combination, Anticandidal mechanism, Kinetics

## Abstract

**Background:**

Treatment of Candida infections have become increasingly difficult due to antifungal drug resistance, which has drawn attention toward the search for innovative and effective drugs. This study aimed to assess the activity of essential oils (EOs) from *Pentadiplandra brazzeana* Baillon (PB) root and *Drypetes gossweileri* S. Moore (DG) stem bark against *Candida albicans* and *Candida parapsilopsis* strains, and determine their antifungal mechanism when tested alone or combined.

**Methods:**

The anticandidal activity of the EOs using the checkerboard format was assessed using the broth micro-dilution technique. The checkerboard microtiter test was performed to evaluate the interaction of the EOs. The in vitro pharmacodynamics of the EOs alone or combined, using time-kill assays, following the chequerboard technique were evaluated. The anticandidal mode of action of these EOs, combined or not, was investigated using the sorbitol protection assay, and the ergosterol binding assay. Differences (*p* < 0.05) between the experimental and the control groups were evaluated using one way analysis of variance (ANOVA) followed by Tukey’s test for multiple comparisons.

**Results:**

Essential oils (EOs) from *Drypetes gossweileri* (DG) stem bark showed activity with MIC value of 62.5 µg/mL against *Candida albicans* and *Candida parapsilopsis*, whereas EOs from *Pentadiplandra brazzeana* (PB) root exhibited MICs of 125 µg/mL and 250 µg/mL against the respective yeasts. The EOs were fungicidal with synergism on *C. parapsilopsis* and additivity on *C. albicans*, with 2 to 64-fold drop in MIC values. The MIC combination of 31.25/7.81 µg/mL and 1.95/31.25 µg/mL (DG/PB EOs) required 20 and 18 h of exposure, respectively to effectively kill 99.9% of the inoculum. This fungicidal effect was accompanied by alteration of the cell walls and membranes of yeasts.

**Conclusion:**

The potency of the EOs combinations indicates further directions in their investigation as potential anticandidal agents.

## Background

*Candida* species are common saprophytic fungi of the human biota in the gastrointestinal tract, and oral and vaginal mucosae. These yeasts frequently colonize human skin and mucosal membranes, thus causing superficial infections such as thrush and vaginitis. However, if the immune defences of the host become compromised, they can cause severe systemic infections, thus contributing significantly to morbidity and mortality. Risk factors for patients include infection by the human immunodeficiency virus (HIV), anticancer therapy, organ transplantation, abdominal surgery, catheters, diabetes, and the prolonged use of broad-spectrum antibiotics [1–4]. Although *C. albicans* (CA) is the prevalent species in candidemia, other species, such as *C. krusei*, *C. glabrata*, *C. tropicalis*, and *C. parapsilosis* (CP), have been observed [5].

The resistance to available antifungals, apart from their severe side effects and lesser efficiency highlight the great need for innovative and effective medicines to treat yeasts infections. An important aspect comprises the search for new compounds that have anti-yeast properties and synergism or additive effect when combined. The advantage of combinational therapy is the greater effect (synergy or additive) of the drugs compared to an individual drug. Similarly, combination of drugs/compounds may result in antagonistic effect. Hence, medicinal plants are used as primary health care in many parts of the world for numerous diseases including candidiasis. Therefore, they can serve as the natural source for the discovery and development of new bioactive medicines. Indeed, the essential oils (EOs) of *Drypetes gossweileri* S. Moore (Euphorbiaceae) stem barks and *Pentadiplandra brazzeana* Baillon (Capparidaceae) roots have been shown to have antioxidant, anti-inflammatory and antimicrobial properties [6–9]. However, to our knowledge, there are no available reports on the underlying kinetics of cell death and mechanism of anticandidal action of *D. gossweileri* and *P. brazzeana* EOs against *C. albicans* and *C. parapsilosis*. Thus, this study is aimed at evaluating the in *vitro* growth inhibition of *D. gossweileri* and *P. brazzeana* EOs both alone and in combination, against *C. albicans* ATCC P37037 and *C. parapsilopsis* ATCC 22,019 strains, and their effects in their growth kinetics, cell wall formation, and ergosterol interactions.

## Materials and methods

### Plant materials and extraction procedure

This study is the continuation of a previous one conducted by Ndoyé Foé et al. in 2016 [9]. The origin of *D. gossweileri* and *P. brazzeana* was indicated and the extraction method was described in that study.In August 2013, *Drypetes gossweileri* stem barks and *Pentadiplandra brazzeana* roots were collected at Awae and Ngomedzap (Center Region of Cameroon), respectively by the sellers. The botanical identification and authentication were carried by Mr. Nana (plant taxonomist) of the National Herbarium of Cameroon (Yaoundé), where voucher specimens were kept: 25,749/SRF/Cam and 42,918/SRF/Cam for *D. gossweileri* and *P. brazzeana* respectively.

The EOs were obtained by hydrodistillation based as in the previous study of Ndoyé Foé et al., 2016, [9]. Briefly, the EOs were extracted by hydrodistillation using a Clevenger-type apparatus for 5 h, dried over anhydrous sodium sulfate and then stored at 4 °C until bioassay. The extraction yields were calculated as the ratio of the mass of EO to the mass of the starting plant material and expressed as a percentage (w/w).

The EOs were analyzed by gas chromatography and gas chromatography coupled to mass spectrometry as described by Agnaniet et al. [10].

### Chemicals and solvents

Ergosterol and D-sorbitol were purchased from Sigma-Aldrich, China and Germany, respectively. Dimethyl sulfoxide (DMSO) was purchased from Sigma-Aldrich, Germany. Fluconazole (Forcan-200, Cipla Pharmaceuticals, India) and ethanol 95% (v/v) were procured from *Pharmacie de l’Université*, in Yaoundé, Cameroon.

### Culture media and microplates

Sabouraud dextrose broth (SDB) and Sabouraud chloramphenicol agar (SDA) were purchased from Titan Biotech Ltd, India, and Fortress Diagnostics Ltd, United Kingdom, respectively. They were prepared and used according to the manufacturers’ instructions. Polystyrene microplates containing 96 wells were purchased from Becton Dickinson and Company, USA.

### Yeast reference strains

The strains used for the study were from the American Type Culture Collection (ATCC), especially *Candida albicans* ATCC P37037 and *Candida parapsilopsis* ATCC 22,019 reference strains provided by BEI Resources NIAID, NIH (Manassas, VA, USA). These yeasts were maintained at room temperature and cultured at 35 °C for 48 h on Sabouraud Dextrose Agar (Oxoid) slants prior to use.

### Anti-yeast activity assay

The Minimum inhibitory concentrations (MIC) were determined by broth microdilution method using the M27-A3 protocol of the Clinical and Laboratory Standards Institute [11], with minor modification: the EOs have not been dissolved in a solvent. Initially, 100 µL of Sabouraud Dextrose Broth (SDB) (Titan Biotech Ltd., India) supplemented with chloramphenicol was distributed in the 96-wells microtiter plates. Then, 100 µL of EO (2000 µg/mL) was transferred to the first well and serially diluted by transferring an aliquot of 100 µL from the first well to the next with EO concentrations ranging from 1000 µg/mL to 7.81 µg/mL, with a geometric connection of ratio 1/2: to pass from a strong concentration at a low, the concentration was multiplied by the factor 1/2. A volume of 100 µL of inoculum suspension at 2.50 × 10^3^ CFU/mL prepared in 0.90% saline, was introduced to each well. Fluconazole was used as positive control, with a starting concentration of 1000 µg/mL. The negative control was: 100 µl of Sabouraud broth, with 100 µl of inoculum. The microtiter plates were incubated at 35 °C in a laboratory incubator for 48 h. A visual reading was performed to determine the MIC of EOs and fluconazole on yeast strains. At the end of the incubation, the lowest test sample concentration with no visible growth, due to the absence of turbidity corresponded to the MIC of the EOs and fluconazole. The test was performed in triplicate.

The Minimum Fungicidal Concentration (MFC) was determined by subculturing 25 μL aliquots of the clear wells into 100 μL of freshly prepared broth medium and incubating at 35 °C in a laboratory incubator for 48 h. The lowest concentration of test sample showing no visible growth was considered as MFC. Wells without inoculum or EOs were included in each plate to control the background sterility and growth. The test was performed in triplicate.

The type of antifungal effect of EOs was deduced from the calculated MFC/MIC ratio, and identified as fungicidal when MFC/MIC ≤ 4, or fungistatic when MFC/MIC > 4 [12].

Based on the anti-yeast activities (MIC, MFC) of *D. gossweileri* and *P. brazzeana* EOs alone and combined, nature of interaction between these EOs was determined by the checkerboard microdilution technique for derivation of the Fractional Inhibitory Concentration Index (FICI) as described below.

### Determination of the interaction of *Drypetes gossweileri* essential oil with *Pentadiplandra brazzeana* essential oil using checkerboard method

A checkerboard microtiter test based on CLSI guideline [11] was performed to evaluate the type of interaction between *D. gossweileri* EO and *P. brazzeana* EO against *C. albicans* ATCC P37037 and *C. parapsilopsis* ATCC 22,019 strains [13]. The series of two-fold dilutions of each oil were made in SDB in the microtiter well. Mixed concentrations in wells ranged from 1/256 × MIC to 2 × MIC for the two EOs. Furthermore, 50 µL of dilution of *D. gossweileri* EO was added to the 96 well microtiter plates in the vertical direction, while 50 µL of dilution of *P. brazzeana* EO was added in the horizontal direction, so that various combinations of EOs could be achieved. Also, 100 µL of yeast inoculum (2.50 × 10^3^ CFU/mL) were added to each well and plates were incubated at 35 °C for 48 h in a laboratory incubator. Each test was performed in triplicate. The nature of interaction was defined quantitatively by means of Fractional Inhibitory Concentrations (FIC) that were calculated as follow: the MIC of the combination of *D. gossweileri* EO with *P. brazzeana* EO divided by the MIC of EO alone. A FIC index (FICI) was obtained by adding both FICs. The combination result was interpreted as follows as described by Van Vuuren and Viljoen [14]: FICI ≤ 0.50, synergistic; > 0.50 to ≤ 1, additive; > 1.00 to ≤ 4.00, indifferent; and > 4.00, antagonistic.

Based on the anticandidal potential of combination of *D. gossweileri* and *P. brazzeana* EOs (synergism and additivity), an insight into the mechanism of anticandidal action was assessed on growth profile, cell wall and cell membrane of *C. albicans* and *C. parapsilopsis* strains, in the presence of EOs alone and in combination.

### Mechanisms of anticandidal action

#### Time-kill kinetic assay

The in vitro pharmacodynamics of *D. gossweileri* and *P. brazzeana* EOs on *C. albicans* ATCC P37037 and *C. parapsilopsis* ATCC 22,019 strains was performed as described by Klepser et al. [15] with some modifications. A volume of 500 µL of an initial inoculum of 1 × 10^5^ CFU/ mL prepared in 0.90% NaCl was seeded onto flat-bottomed 24-well microtitration plates, with 400 μL of SDB and 100 μL of each EO alone. For combination of EOs, same volume and concentration of inoculum was seeded, with 300 μL of SDB and 100 μL of each sample of EO of the combination. The plate was incubated at 35 °C under orbital shaking, 32 × g (IKA-Vibrax-VXR, Radnor, PA, USA) at various time periods (0, 2, 4, 6, 8, 10, 12, 14, 16, 18, 20 and 24 h). At predetermined time points, 10 μL of the mixture was pipetted and diluted in 40 μL normal saline onto flat-bottomed 96-well microtitration plates. The dilutions were homogenized for the determination of viable colony counts using a Malassez counting cells (Thermo Fisher Scientific, Darmstadt, Germany) and expressed in log_10_ CFU/mL. The experiment was performed in duplicate. Time-kill curves were constructed by plotting mean of colony count (log_10_ CFU/mL) as a function of time (hours) for each time point. The effect of the EOs was considered fungicidal when there was a decrease greater than or equal to 3 log_10_ CFU/mL of the initial inoculum, resulting in reduction of 99.9% or more CFU/mL in 24 h compared with the initial inoculum. Fungistatic activity was considered as reduction in growth lower than 99.9% or < 3 log_10_ in CFU/mL from the initial inoculum [14]. The criteria used to interpret the interactions between EOs: Synergism was obtained when the fungicidal effect led to ≥ 2 log_10_ decrease in cells/mL for the combination compared to the most active EO; additivity was defined as < 2log_10_ decrease in cells/mL for the combination compared to the most active EO; indifference as < 2 log_10_ increase in cells/mL for the combination compared to the least active EO; and antagonism as ≥ 2log_10_ increase in cells/mL for the combination compared to the least active EO [15].

The mode of action of EOs alone and in combination was also performed to determine whether the anticandidal activity found is the result of a direct interaction with the cell wall structure of *Candida* strains (sorbitol protection assay) or the ion permeability of their membrane (ergosterol effect assay).

#### Sorbitol protection assay

The MICs of *Drypetes gossweileri* and *Pentadiplandra brazzeana* EOs in the presence sorbitol (an osmotic protector) against Candida strains were determined using the microdilution technique [11] in triplicate.

Initially, 100 µL of SDB was introduced into each well of the microplate. Subsequently, 100 µL of EOs solutions were transferred to the first well and serially diluted two-fold dilution. *D. gossweileri* and *P. brazzeana* EOs concentrations ranged from 1000 µg/mL to 15.63 µg/mL and from 2000 µg/mL to 31.25 µg/mL respectively. For EOs in combination, concentrations ranged from 500 µg/mL to 7.81 µg/mL were obtained. Then, 50 µL of yeast inoculum (2.50 × 10^3^ CFU/mL) prepared in SDB and 50 µL of sorbitol (Sigma-Aldrich, USA) were transferred to the wells for a final concentration of 0.80 M sorbitol in each well [16, 17]. The negative control included 100 µL of SDB and 50 µL of the inoculum with 50 µL of sorbitol (0.80 M) in each cavity. Sterility control was also performed: 100 µL SDB with 50 µL of sorbitol (0.80 M) was placed in a plate column without fungal suspension. The plates were incubated at 35 °C in a laboratory incubator, and the results were read after 48 h [16, 17]. MIC was determined as the lowest concentration of test EOs inhibiting the visible growth. Each experiment was repeated three times and mean values were calculated for MICs.

#### Ergosterol effect assay

First, 100 µL of SDB was added to each well of the microplate. Then, 100 µL of EOs solutions were transferred to the first well and serially diluted by transferring a 100 µL aliquot from the most concentrated well to the next well with *D. gossweileri* and *P. brazzeana* EOs concentrations ranged from 1000 µg/mL to 15.63 µg/mL and 2000 µg/mL to 31.25 µg/mLrespectively Concerning EOs in combination, EOs concentrations ranged from 500 µg/mL to 7.81 µg/mL. A volume of 50 µL of yeast inoculum (2.50 × 10^3^ CFU/mL) prepared in SDB and 50 µL of ergosterol (Sigma-Aldrich, China) were transferred to the wells for a final concentration of 250 µg/mL ergosterol in each well. The plates were incubated at 35 °C in a laboratory incubator, and the results were read after 48 h [17, 18]. Yeast growth and sterility were also controlled. Fluconazole was tested as a positive control. MIC was determined as the lowest concentration of test EOs inhibiting the visible growth. Each experiment was repeated three times and mean values were calculated for MICs.

### Statistical analysis

The results were the means of concentrations ± standard deviations from triplicate values obtained from three independent experiments.The data were statistically analysed using the software SPSS 17.0 for Windows and analysis of variance (ANOVA) coupled with Tukey test. A *p* < 0.05 was considered as statistically significant.

## Results

### Anticandidal activity of essential oils alone and in combination against *Candida* strains

The Minimum Inhibitory Concentration (MIC) and Minimum Fungicidal Concentration (MFC) Minimum Fungicidal Concentration (MFC) values of *Drypetes gossweileri*, *Pentadiplandra brazzeana* essential oils and fluconazole against *C. albicans* ATCC P37037 and *C. parapsilopsis* ATCC 22,019 are shown in Table 1.

**Table 1:** MIC and MFC of *DG*, *PB* EOs and fluconazole on *CA* and *CP* strains

	***C. albicans* ATCC P37037**			***C. parapsilopsis* ATCC 22019**		
	MIC	MFC	MFC/MIC	MIC	MFC	MFC/MIC
*D. gossweileri *EO MIC (µg/mL)	62.50 ± 0*	62.50 ± 0*	1	125 ± 0	125 ± 0	1
*P. brazzeana* EO MIC (µg/mL)	62.50 ± 0*	62.50 ± 0*	1	250 ± 0	250 ± 0	1
Fluconazole	15.63 ± 0***	31.25 ± 0**	2	62.50 ± 0*	125 ± 0	2

Legend: * indicate *P* ≤ 0.05., ** *P* ≤ 0.01., *** *P* ≤ 0.001

The results of the study by broth microdilution showed that the MICs of *D. gossweileri* EO were obtained at 62.50 µg/mL for *C. albicans* and 125 µg/mL for C*. parapsilopsis*. The MICs of *P. brazzeana* EO were 62.50 µg/mL for *C. albicans* and 250 µg/mL for C*. parapsilopsis*. The MFC values of each EO, except for fluconazole were the same with MICs against *Candida* strains. The ratio MFC/MIC values were 1 and 2 for both essential oils and fluconazole, respectively.

Having established the individual MIC and MFC values, the MIC and Fractional inhibitory concentrations index (FICI) values of *D. gossweileri* stem barks EO and *P. brazzeana* roots EO in combination were determined using checkerboard assays against the same yeast strains, in the aim of the detection of synergism, additivity or antagonism between these EOs. The results of the FICI are shown in Table 2.

**Table 2:** MIC, FIC and FICI of *DG* EO combined with *PB* EO against *CA* and *CP* strains

	***C. albicans* ATCC P37037**	***C. parapsilopsis* ATCC 22019**
*D. gossweileri *EO MIC (µg/mL)	31.25*	1.95***
*P. brazzeana* EO MIC (µg/mL)	7.81**	31.25*
FIC index (FICI)	0.63	0.14
FICI effect	Additivity	Synergism

Legend: * indicate *P* ≤ 0.05., ** *P* ≤ 0.01., *** *P* ≤ 0.001

Based on the anticandidal potential of combination of *D. gossweileri* EO with *P. brazzeana* EO (synergistic and additive), an insight into the growth profile of *C. albicans* ATCC P37037 and *C. parapsilopsis* ATCC 22,019 strains, through the time-kill studies were performed over a period of 24 h.

### Time-kill kinetics of essential oils alone and in combination for *Candida* strains

**Fig. 1:**
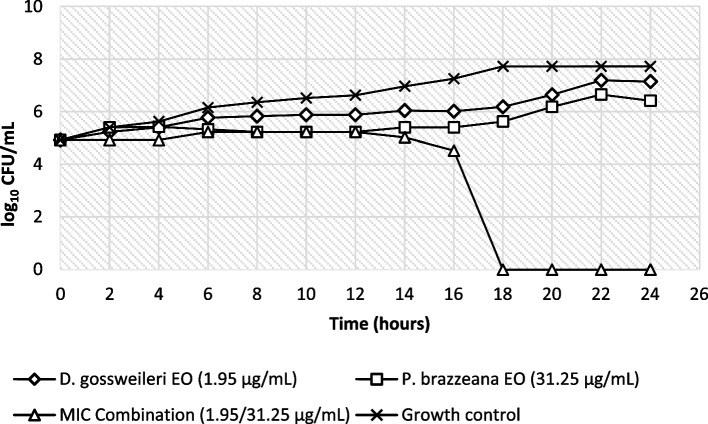
Time kill curve of *DG* and *PB* EOs in combination on *Candida parapsilopsis* ATCC 22019

**Fig. 2:**
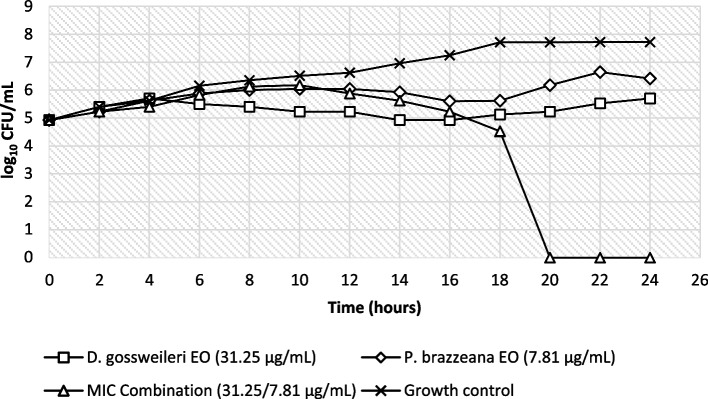
Time kill curve of *DG* and *PB* EOs in combination on *Candida albicans* P37037

The results of the time-kill curves for *C. parapsilopsis* ATCC 22,019 and *C. albicans* ATCC P37037 being exposed to MIC values (EOs combination) and sub-MIC values (EO alone) of *D. gossweileri* and *P. brazzeana* are shown in Figs. 1 and 2.

The growth profile of *Candida* strains in the presence of *D. gossweileri* and *P. brazzeana* EOs was assessed to further corroborate the in vitro anticandidal activity results observed above.

For the combination of *D. gossweileri* EO with *P. brazzeana* EO against *C. albicans* strains, a significant reduction in the colony count was recorded between 18 and 24 h, whereas for *C. parapsilopsis* strains, it was between 16 and 24 h. The analysis of the log_10_ CFU/mL versus time graph shows that the fungicidal activity against *Candida albicans* ATCC P37037 was achieved after 20 h at MIC combination of *D. gossweileri*/*P. brazzeana* EOs (31.25/7.81 µg/mL), while for *C. parapsilopsis* ATCC 22,019, after 18 h at MIC combination *D. gossweileri*/*P. brazzeana* EOs (1.95/31.25 µg/mL). *C. parapsilopsis* strains were the most susceptible to MIC combination, with all cells killed within 16 h of exposure.

The combination of EOs studied in this paper demonstrated fungicidal potential within 24 h, emphasizing their potential as source of anti-yeast agents with mode of action to be investigated.

### Mode of anticandidal action of essential oils alone and in combination

The elucidation of the antifungal mode of action of *Drypetes gossweileri* stem barks and *Pentadiplandra brazzeana* roots EOs, alone and in combination were investigated through sorbitol protection assay and ergosterol effect assay, whose results are presented in Tables 3 and 4, respectively.

**Table 3:** Sorbitol effect on EOs MIC alone and in combination for *CA* and *CP* strains

Sorbitol in the media (mol/L)	MIC (µg/mL)			
		*D. gossweileri *EO (A)	*P. brazzeana* EO (B)	Combination A/B
**0.0**	*C. albicans* ATCC P37037	62.5*	62.5*	> 31.25/7.81
	*C. parapsilopsis* ATCC 22019	125	250	> 1.95/31.25
**0.8**	*C. albicans* ATCC P37037	1000	1000	> 500/125
	*C. parapsilopsis* ATCC 22019	500	500	> 31.25/500

Legend: * indicate * *P* ≤ 0.05

**Table 4:** Exogenous ergosterol effect on EOs MIC alone and in combination for *CA* and *CP* strains

**Ergosterol in the media (µg/mL)**	**MIC (µg/mL)**				
		*D. gossweileri* EO (A)	*P. brazzeana* EO (B)	Combination A/B	Fluconazole
**0.0**	*C. albicans* ATCC P37037	62.5*	62.5*	> 31.25/7.81	15.63
	*C. parapsilopsis* ATCC 22019	125	250	> 1.95/31.25	125
**250**	*C. albicans* ATCC P37037	500	500	> 500/125	125
	*C. parapsilopsis* ATCC 22019	500	1000	> 31.25/500	500

Legend: * indicate * *P* ≤ 0.05

In this paper, it was found that the *P. brazzeana* and *D. gossweileri* EOs MICs against *C. albicans* ATCC P37037 and *C. parapsilopsis* ATCC 22,019 strains increased by 4 to 16-fold in the presence of sorbitol. When the yeasts were treated with EOs combination in a medium supplemented with sorbitol, MIC values did shift to higher values. As can be seen, the results indicated that the mechanism of action of EOs tested alone or combined act by inhibiting fungal cell wall synthesis.

Based on the results of the present study, the MIC values of *D. gossweileri* EO against *C. albicans* and *C. parapsilopsis* increased eight (62.50 μg/mL to 500 μg/mL) and four times (125 μg/mL to 500 μg/mL), respectively, in the presence of exogenous ergosterol (Table 3). The same was also observed for *P. brazzeana* EO against *C. albicans* (62.50 μg/mL to 500 μg/mL) and *C. parap*silopsis (250 μg/mL to 1000 μg/mL). In addition, the MIC values of combination of *P. brazzeana* and *D. gossweileri* EOs against *C. albicans* and *C. parapsilopsis* increased up to sixteen (> 31.25/7.81 μg/mL to > 500/125 μg/mL) and sixteen/four times (> 1.95/31.25 μg/mL to > 31.25/500 μg/mL), respectively. The results indicated that the mechanism of action of the EOs involves a primary lesion of the cell membrane, leading to cell death.

## Discussion

### Anticandidal activity of essential oils alone and in combination against *Candida* strains

*C. albicans* ATCC P37037 strains were more sensitive than *C. parapsilopsis* ATCC 22,019 to EOs and fluconazole. According to criteria (MFC/MIC < 4) proposed by Carbonnelle et al. [11], *D. gossweileri*, *P. brazzeana* EOs and fluconazole showed fungicidal activities against the two ATCC yeasts. To be fungicidal rather than fungistatic is an important finding since antifungal agents that kill fungi (cidal) have demonstrated to be, in most cases, clinically more useful than those that merely inhibit (static) fungal growth [18]. In literature, *P. brazzeana* roots EOs have been found to be active against yeast and filamentous fungal species [8; 9]. Indeed, Nyegue et al. [7] found that *P. brazzeana* EO was two-fold more active against *C. albicans* than this found in the present study, with MIC and MFC values of 31.25 µg/mL. This could be due to the difference in chemical composition within these essential oils [9]).

The Fractional Inhibitory Concentrations Index (FICI) calculated from the results of the checkerboard assay revealed the following: the investigation of antifungal activity of *D. gossweileri* EO in combination with *P. brazzeana* EO against *C. albicans* and *C. parapsilopsis* caused a significant decrease in the MIC, compared to their individual MIC values. The MIC of *D. gossweileri* EO alone against *C. parapsilopsis* was lowered from 125 µg/mL to 1.95 µg/mL, so a 64-fold reduction, in the presence of *P. brazzeana* EO. The MIC of *P. brazzeana* EO alone also decreased from 250 to 31.25 mg/mL, so an eightfold reduction. Thus, for *C. parapsilopsis*, this combination was classified as synergetic, with a FIC index of 0.14. For the strains of *C. albicans*, the MIC value of *D. gossweileri* EO alone was 62.50 µg/mL. When associated with *P. brazzeana* EO, a twofold reduction in the MIC value (62.50 µg /mL to 31.25 µg/mL) of the same EO was observed. An eightfold reduction in the MIC value (62.50 µg /mL to 7.81 µg/mL) of *P. brazzeana* EO was also observed when associated with *D. gossweileri* EO. Thus, this association was classified as additive, with an FIC index of 0.63. These observations highlight antifungal potential of combination therapy using *D. gossweileri* EO with *P. brazzeana* EO against *C. albicans* and *C. parapsilopsis* strains.

### Time-kill kinetics of essential oils alone and in combination for *Candida* strains

The growth profile of *Candida* strains in the presence of *Drypetes gossweileri* and *Pentadiplandra brazzeana* EOs was assessed to further corroborate the in vitro anticandidal activity results observed.

The time of death curves showed that both EOs tested alone at sub-MIC were fungistatic against the two *Candida* strains. On the contrary, the combination of *D. gossweileri* and *P. brazzeana* EOs at MIC exhibited fungicidal activity against the tested yeasts. The combination of *D. gossweileri* and *P. brazzeana* EOs recorded significant reduction in the CFU/mL over the time when compared to the effect of individual EO. Indeed, to promote greater efficiency of *D. gossweileri* and *P. brazzeana* EOs, when used at lower concentrations, the association of these EOs could be proposed.

Besides, synergistic and additive effects observed in checkerboard microdilution were confirmed by time-killing assay. The time kill characterization is very important because it has valuable therapeutic implications, such as adjusting the dose for a more effective treatment [18] or shorten the duration of therapy and avoid the emergence of resistance to available antifungals [19, 20]. To the best of our knowledge, the kinetics of *D. gossweileri* and *P. brazzeana* EOs on *C. albicans and C. parapsilosis* cells death remain unknown. Also, it is noteworthy that this is the first study on the aspect of optimizing anti-*Candida* activity by coupling *D. gossweileri* and *P. brazzeana* EOs in combination. These anti-*Candida* activities of *D. gossweileri* and *P. brazzeana* EOs and their optimization in combination is probably due to the combined effect of all components of both essential oils (as indicated in a previous report by Ndoyé et al. [9]): terpenes, sulfur- and nitrogen-containing compounds, acting synergistically and additively against the targeted *Candida* strains.

### Mode of anticandidal action of essential oils alone and in combination

The sorbitol assay consisted of determining the MIC in the presence and absence of 0.8 M sorbitol, an osmotic protector used to stabilize fungi protoplasts. Cells protected with sorbitol can grow in the presence of fungal cell wall inhibitors, whereas growth would be inhibited in the absence of sorbitol. This effect is detected by increase in the MIC value as observed in medium with sorbitol as compared to the MIC value in medium without sorbitol (standard medium) [16, 21].

The ergosterol effect assay consisted of determining whether D. gossweileri and P. brazzeana EOs bind to the membrane sterols of tested yeasts. If the activity of EOs was caused by binding to ergosterol, the exogenous ergosterol would prevent the binding to ergosterol in the membranes of yeasts. Consequently, MIC increase for EOs (in the presence of exogenous ergosterol in relation to the control assay) would occur because only increased EOs concentration in the growth medium might assure interaction with ergosterol in the membranes of yeasts [22, 23]. Thus, the effect of exogenous ergosterol on EOs and fluconazole MIC was determined.

The findings of this study suggest that *Pentadiplandra brazzeana* and *Drypetes gossweileri* EOs alone act by altering the structure of the cell wall and cell membrane of yeast. When combined, it could be expected that *P. brazzeana* and *D. gossweileri* EOs could enhance their permeability to fungi by altering fungal cell wall and membrane integrity that may intensify the fungal killing. However, the cascades of multiple secondary effects such as reactive oxygen species (ROS) accumulation, mitochondrial membrane potential dissipation, and DNA condensation and fragmentation (remain to be established in studies that are out of scope for this paper) as a result of membrane disruption action cannot be overlooked as a cause of death.

The elucidation of the action mechanisms of *P. brazzeana* and *D. gossweileri* EOs is another strategy which require further detailed investigations for limiting the emergence of resistance to the currently available antifungal agents, as well as for developing rational, safer and more potent alternative therapies against *Candida* infections which frequently require combinations of drugs or the use of new drugs when the first-choice agent is not effective. It is noteworthy that from our literature review, there is no scientific report on investigation of possible action of *P. brazzeana* and *D. gossweileri* EOs on cell wall and cell membrane, as primary action mechanism. Drugs that act on the cell wall cause lysis of fungal cells in the absence of an osmotic stabilizer (sorbitol), but their growth can continue in the presence of sorbitol [22]. Inhibition of growth is detected by increase in the MIC values as observed in medium with sorbitol as compared to the MIC value in medium (standard medium) without sorbitol [16]. According to Frost et al. [16], this assay is generic in nature and is of use in the search of substances that directly inhibit the synthesis of cell wall constituents such as glycans, mannans or chitin as found in this study of the effect of *P. brazzeana* and *D. gossweileri* EOs on cell wall.

The ability of *P. brazzeana* and *D. gossweileri* EOs to form complexes with ergosterol was evaluated from the perspective of investigating their action on the yeasts cell membrane. It was found that the EOs bind to exogenous ergosterol, avoiding it to ergosterol membrane binding. The results of this study suggest that *P. brazzeana* and *D. gossweileri* EOs appear to bind to the ergosterol in the membrane, promoting increased membrane permeability, or inactivated plasma membrane-ATPase (an important fungal pump which transfers substances in fungal plasma membrane), causing the depletion of components essential to fungal cell survival and ultimately cell death. It seems that antifungal mechanism of action of these EOs is similar to fluconazole. Fluconazole is a triazole which mainly act through the inhibition of lanosterol-14-alpha-demethylase, a key enzyme involved in the biosynthesis of ergosterol, an important component of the fungal cell membrane [24].

Damage to cell wall and cell membrane by *P. brazzeana* and *D. gossweileri* EOs is probably due to the combined effect of all components of both oils: terpenes, sulfur- and nitrogen-containing compounds, acting synergistically and additively against tested yeasts. Indeed, it was reported that the antifungal mechanisms of action for the nitrogen containing compounds are mainly a result of cell membrane disruption via inhibition of ergosterol biosynthesis, or complexing with ergosterol [25, 26]. Also, Souza et al. [26] reported that the sulfur compounds interfere with membrane integrity or associated enzyme proteins, stopping their production or activity.

Similarly, the simultaneous actions of EOs components on different targets enhance their bioactivity and might also reduce the advent of resistance by the fungi. Generally, the chemical configuration of terpenes gives them hydrophobic properties and allows them to deposit on the lipophilic structures of microorganisms such as the plasma membrane; this deposition leads to increased permeability with a consequent loss of the electrolytes essential to cell survival [27]. In line with this, Nguefack et al. [28] showed that the carbohydrates terpenes, although having a lesser activity, can allow rupture of the cell membrane, thus promoting the intracellular transport of antimicrobial compound such as sulfur derivatives present in both EOs. Indeed, isothiocyanate derivatives are known for their strong antimicrobial activity. Their isothiocyanate group (R-N = C = S), has a strongly electrophilic carbon that can easily react with a nucleophilic center, and cleaving the disulfide bonds of the proteins and degrading amino acids via oxidation reactions [29, 30] with production of reactive oxygen species (ROS). Therefore, free radicals oxidize and damage lipids, proteins and DNA. Moreover, some phenolic components of essential oils are oxidized by contact with ROS producing very reactive phenoxyl radicals which add to the ROS released by mitochondria. These types of radical reactions are dependent on and enhanced by the presence of cell transition metal ions [31, 32]. The anticandidal activity of combined EOs can promote greater efficiency of *D. gossweileri* and *P. brazzeana* EOs when used at lower concentrations, allowing synergistic and additive effects with 2 to 64-fold reduction of the concentrations of both essential oils in relation to the effect provided when assessed separately.

## Conclusion

This study assessed the antifungal activity, kinetics of cell death and mode of action of essential oils (EOs) from *Pentadiplandra brazzeana* roots and *Drypetes gossweileri* stem barks, alone and in combination, against *C. albicans* ATCC P37037 and *C. parapsilopsis* ATCC 22,019 strains. The findings showed that EOs from *P. brazzeana* roots and *D. gossweileri* stem barks were fungicidal, alone and combined, with synergism on *C. parapsilopsis* and additivity on *C. albicans*, with 2 to 64-fold drop in MIC values after 18 h and 20 h, respectively. Furthermore, fungicidal effect of EOs alone and in combination was accompanied by the disturbance of cell wall and ergosterol biosynthesis in *C. albicans* cells. The potency of the EOs combination might be mainly beneficial to treat candidiasis. Meanwhile, further investigations as potential anticandidal agents will be necessary to elaborate more knowledge about other *Candida* cell targets with respect to EOs from *P. brazzeana* roots and *D. gossweileri* stem barks.

## Data Availability

The datasets used and analysed during the current study are available from the corresponding author on reasonable request.

## Abbreviations

ATCC: American Type Culture Collection
CFU/mL: Colony Forming Units per millilitre
DMSO: Dimethyl sulfoxide
DG: *Drypetes gossweileri* S. Moore
EO: Essential Oils
FICI: Fractional Inhibitory Concentrations Index
GC/MS: Gas Chromatography-Mass Spectrometry
MIC: Minimum Inhibitory Concentration
MIF: Minimum Fungicidal Concentration
PB: *Pentadiplandra* *brazzeana* Baillon
SDA: Sabouraud Chloramphenicol Agar
SDB: Sabouraud Dextrose Broth
w/w: Weigh/weigh
